# Revised genomic structure of the human ghrelin gene and identification of novel exons, alternative splice variants and natural antisense transcripts

**DOI:** 10.1186/1471-2164-8-298

**Published:** 2007-08-30

**Authors:** Inge Seim, Chris Collet, Adrian C Herington, Lisa K Chopin

**Affiliations:** 1 Institute of Health and Biomedical Innovation, Queensland University of Technology, Kelvin Grove, Queensland, Australia

## Abstract

**Background:**

Ghrelin is a multifunctional peptide hormone expressed in a range of normal tissues and pathologies. It has been reported that the human ghrelin gene consists of five exons which span 5 kb of genomic DNA on chromosome 3 and includes a 20 bp non-coding first exon (20 bp exon 0). The availability of bioinformatic tools enabling comparative analysis and the finalisation of the human genome prompted us to re-examine the genomic structure of the ghrelin locus.

**Results:**

We have demonstrated the presence of an additional novel exon (exon -1) and 5' extensions to exon 0 and 1 using comparative in silico analysis and have demonstrated their existence experimentally using RT-PCR and 5' RACE. A revised exon-intron structure demonstrates that the human ghrelin gene spans 7.2 kb and consists of six rather than five exons. Several ghrelin gene-derived splice forms were detected in a range of human tissues and cell lines. We have demonstrated ghrelin gene-derived mRNA transcripts that do not code for ghrelin, but instead may encode the C-terminal region of full-length preproghrelin (C-ghrelin, which contains the coding region for obestatin) and a transcript encoding obestatin-only. Splice variants that differed in their 5' untranslated regions were also found, suggesting a role of these regions in the post-transcriptional regulation of preproghrelin translation. Finally, several natural antisense transcripts, termed ghrelinOS (ghrelin opposite strand) transcripts, were demonstrated via orientation-specific RT-PCR, 5' RACE and in silico analysis of ESTs and cloned amplicons.

**Conclusion:**

The sense and antisense alternative transcripts demonstrated in this study may function as non-coding regulatory RNA, or code for novel protein isoforms. This is the first demonstration of putative obestatin and C-ghrelin specific transcripts and these findings suggest that these ghrelin gene-derived peptides may also be produced independently of preproghrelin. This study reveals several novel aspects of the ghrelin gene and suggests that the ghrelin locus is far more complex than previously recognised.

## Background

Ghrelin is a 28 amino acid peptide hormone originally isolated from the stomach (where it is highly expressed) and it is the endogenous ligand for the growth hormone secretagogue receptor (GSH-R 1a) [1]. It is well established that ghrelin is a multifunctional peptide with roles in growth hormone release, appetite regulation and gut motility [2] and we have demonstrated that it plays a role in cancer cell proliferation [3-5]. Despite its widespread and important physiological actions, its precise regulatory mechanisms remain ambiguous. Compared to other preprohormones, the genomic structure of ghrelin is thought to be relatively simple, consisting of four coding exons and a short, 20 bp first exon [6,7], hereafter termed exon 0. The ghrelin gene (*GHRL*) spans 5 kb on chromosome 3 [6-8] and exons 1 to 4 encode an 117 amino acid preprohormone, preproghrelin. The preproghrelin signal peptide is encoded in exon 1, and the coding sequence of the 28 amino acid ghrelin peptide hormone is encoded by parts of exons 1 and 2. Exon 3 codes for obestatin, a recently identified 23 amino acid ghrelin gene-derived peptide hormone [9]. The physiological relevance of obestatin is somewhat controversial, as it does not circulate in human serum, although the C terminal peptide of ghrelin, C-ghrelin does [10]. C-ghrelin, encoded by exons 2, 3 and 4, is a 66 amino acid peptide that contains the 23 amino acid obestatin peptide within its sequence [10,11]. It is currently not known if obestatin is cleaved from the large preproghrelin peptide, or whether distinct human obestatin-only and C-ghrelin-only transcripts exist. We have previously reported an obestatin-deleted transcript [4]. Interestingly, a murine intron 1 retained variant lacking exon 0, 3 and 4 has recently been reported [12]. The transcript therefore lacks the coding sequence of obestatin, but contains a putative peptide containing the first five amino acids of ghrelin and a novel 19 amino acid sequence.

Re-examination of the ghrelin locus is required for a number of reasons. First, the ghrelin gene structure has not been examined since the finalisation of the human chromosome 3 sequence in 2006 [13] and the release of orthologous sequencing data. Second, newly developed bioinformatic tools now enable comparative genomics analyses. The aim of this study was, therefore, to re-examine the organisation of the human ghrelin gene with the aid of recently available genomic sequence information from multiple species, including the continuously updated draft mouse [14] and chicken [15] genomes. Using *in silico *approaches we predicted the existence of a novel, distal ghrelin exon (exon -1) and this was confirmed experimentally using 5' RACE and RT-PCR. We have also identified the expression of extended exon 0 species and re-annotated a 5' extended exon 1 not previously recognised in the literature [6]. Multiple alternative mRNA transcripts were also identified experimentally from normal tissues and from prostate cell lines and a chondrosarcoma cell line, indicating that the ghrelin gene has a complex transcriptional pattern. In addition, we report a gene on the antisense strand of the ghrelin gene, ghrelinOS (ghrelin opposite strand), and have demonstrated the expression of endogenous natural antisense transcripts (NATs) that partially overlap the recognised sense ghrelin gene exons.

## Results and discussion

### Conserved regions identified using comparative genomic analysis of the ghrelin gene are transcribed

**Figure 1:**
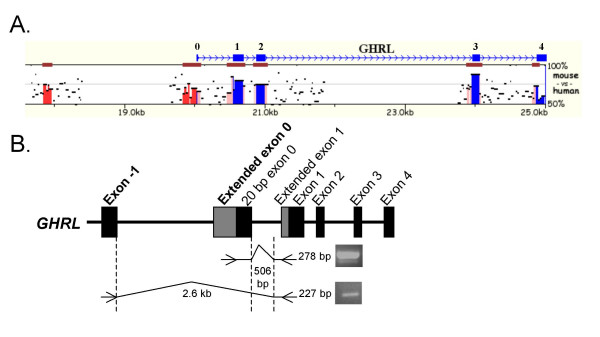
**Initial identification and verification of novel transcribed ghrelin regions using comparative analyses and RT-PCR. A**. Mulan sequence conservation profile for the human and murine ghrelin loci. The horizontal axis displays the input sequence. Evolutionary conserved regions (ECRs, > 70% identity; ≥ 99 bp) are depicted as dark red bars above each pairwise alignment. Preproghrelin coding exons 1 to 4 and the non-coding 20 bp exon 0 (blue), intergenic elements (red) and intron sequence (pink) are marked and the vertical axis shows the percent similarity of the murine ghrelin orthologue to the human sequence. Two conserved intergenic regions, 506 and 2.6 kb upstream of exon 1 of the ghrelin gene, can be seen in red. **B**. Schematic diagram showing the location of RT-PCR primers employed to verify whether the conserved, intergenic regions identified by Mulan were transcribed from the ghrelin gene. A forward primer in the conserved region immediately upstream of the 20 bp exon 0 and a reverse primer in exon 1 amplified a 278 bp PCR fragment (upper panel). A PCR using another exon 1 primer and a forward primer in the conserved region 2.6 kb upstream of exon 1 resulted in a 227 bp PCR fragment (lower panel). The PCRs confirm that the conserved regions predicted by Mulan correspond to ghrelin gene-derived exons. We have termed the conserved regions exon -1 and extended exon 0.

Mulan analysis of the human and mouse ghrelin loci revealed the presence of two conserved regions (> 70% identity and ≥ 99 bp) (Fig. 1A). Neither of these upstream regions was conserved in the draft chicken genome sequence, even when the alignment stringency was altered (data not shown). The 3' terminal 20 bp of a ~200 bp conserved region is equivalent to a short, non-coding exon 0 sequence (506 bp upstream of exon 1) previously demonstrated in human [6,7] and in murine [16] ghrelin transcripts, suggesting the existence of transcripts with an exon 0 that is considerably larger than the previously reported 20 bp exon 0. A second, distal ~100 bp region approximately 2.6 kb upstream from exon 1 is also conserved.

### The conserved regions upstream of exon 1 are ghrelin exons

To examine whether the conserved regions identified using Mulan were transcribed, PCRs from human stomach (using cDNA reverse transcribed with oligo(dT)_18 _primers) were performed with antisense primers in exon 1 of ghrelin and sense primers in the conserved regions. Agarose gel electrophoresis of these PCRs showed that both of the conserved regions were transcribed (Fig. 1B) and their identity was confirmed by sequencing. The putative exon regions appeared to be mutually exclusive in the human stomach and the putative exon-intron junctions conform to the GT/AG rule. Therefore, initial analysis indicated that the conserved regions are true exons and not artefacts. The two conserved regions have been termed exon -1 [GenBank:EF549566] and exon 0 [GenBank:EF549567]. In addition to these two regions, a sequence immediately upstream of exon 1 of the human ghrelin gene is conserved in comparison to mouse sequence (Fig. 1A). This conserved sequence matches the first 50 base pairs of a 188 bp exon 1 sequence previously reported in mRNAs isolated from human stomach and thyroid medullary carcinoma TT cells [6]. The previous study, however, did not annotate this sequence as a 5' extended exon 1 (and we have termed it exon 1b). We have, therefore, identified a novel, distal exon and 5' extensions to two previously reported exons (20 bp exon 0 and exon 1).

### Exon 0 and -1 are novel first exons

**Figure 2:**
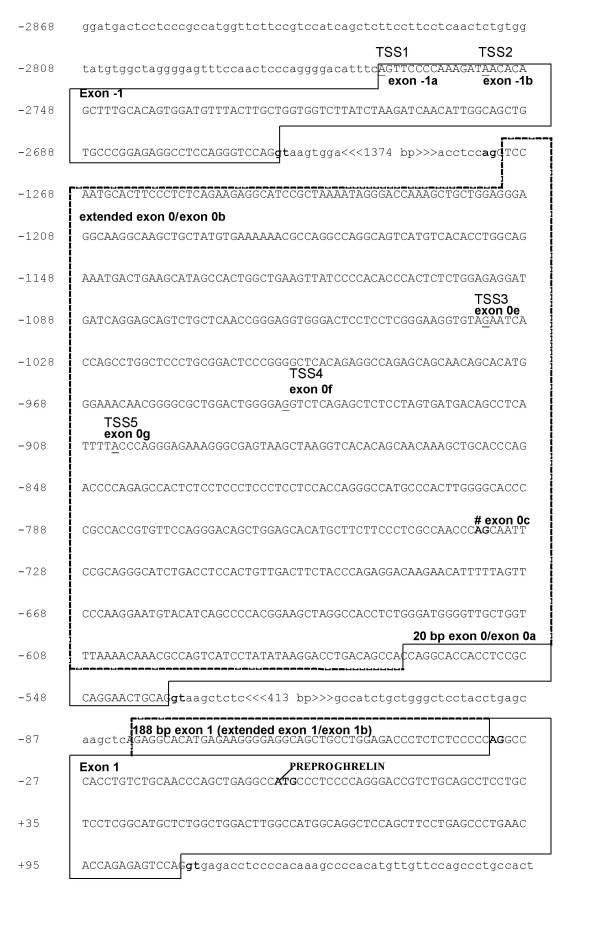
**Partial genomic sequence of the human ghrelin gene showing novel exon -1 and extended exon 0 transcription start sites**. Exon sequences are boxed and in upper case letters. Extended exon 0 sequence is indicated by dashed boxes. Nucleotide positions are shown on the left and the translation initiation site of preproghrelin is indicated as +1. Intron boundaries are indicated by bold, and intron sequences by lower case letters. Five transcription start sites (TSS) determined by employing an OriGene human Sure-RACE panel, denoted TSS1-5, are underlined and indicated. TSS1 contains a 106 bp exon -1 (exon -1a) and splices into the complete, 736 bp 5' extended exon 0 (exon 0b) [GenBank:EF549561]. TSS2 [GenBank:EF549564] harbours a 92 bp exon -1 (exon -1b) and splices into a 197 bp exon 0 (exon 0c; exon acceptor site is indicated # and bold). TSS3-5 all initiate in exon 0 (exon 0 e-g, respectively). TSS3 was obtained from the ovary [GenBank:EF549563]; TSS4 from testis [GenBank:EF549562] and stomach; TSS5 from adipose tissue [GenBank:EF549565] and leukocytes.

To examine if the two novel, putative exon -1 and extended exon 0 regions were first exons, that is if transcription start site(s) (TSS) were present, 5' RACE was performed. Exon 0-specific reverse primers and a RACE-ready panel of anchored cDNA libraries derived from 24 human tissues (OriGene, Rockville, MD) were used. Sequencing of the 5' RACE clones from two replicate, independent experiments identified two exon -1 start sites, as well as multiple transcription start sites in exon 0 (Fig. 2). The cDNA sequences of the 5' RACE [GenBank:EF549561, EF549562, EF549563, EF549564, and EF549565] products are shown in Fig. 2. The longest 5' RACE clone identified (TSS1 in Fig. 2) is expressed in adipose tissue, leukocytes and the uterus [GenBank:EF549561] and contains a 106 base pair exon -1 and a large 736 bp exon 0 (exon 0b, the complete extended exon 0 shown in Fig. 2).

**Figure 3:**
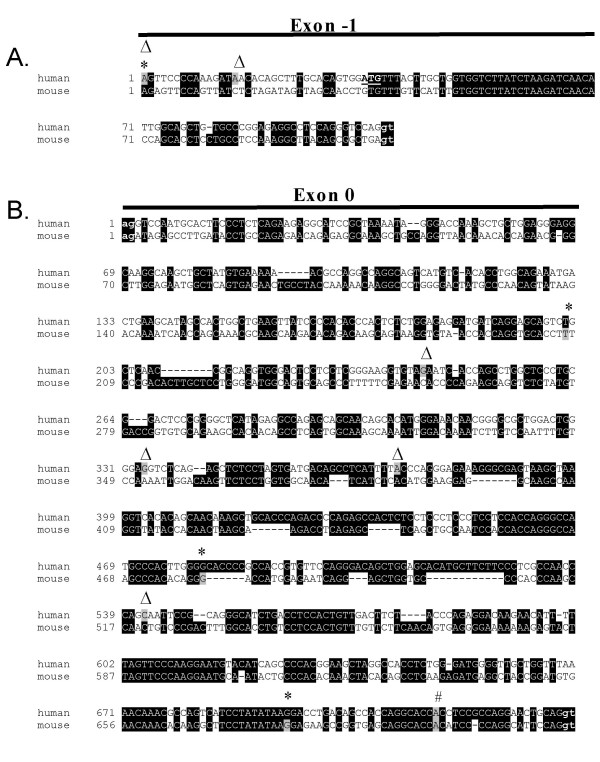
**Comparison of novel human and putative murine ghrelin exon sequences**. The mouse and human alignments were generated by the ClustalW program and drawn by BOXSHADE . Black shading indicates conserved nucleotides. Transcription start sites determined by 5' RACE (Δ) and CAGE (Cap Analysis of Gene Expression) (*) are indicated, and the exact transcription start site nucleotides are shaded in grey in each species. Exon-intron boundaries are indicated by lowercase letters and bold font. **A**. Comparison of human and putative mouse transcription start sites (TSSs) in the novel exon -1. Two human TSSs are indicated, corresponding to 106 bp (5' RACE and CAGE tag starting site T03R009D4FFF) and 92 bp exon -1. An initiating methionine (ATG, start codon) which is not followed by a stop codon in exon -1 is underlined. No murine exon -1 CAGE tags were found in the FANTOM3 Basic CAGE viewer (May 2007) **B**. Comparison of human and mouse extended exon 0 sequences showing several human and murine exon 0 transcription start sites deduced by 5' RACE and CAGE (mouse T06R06CF33DC, T06R06CF32CB and T06R06CF3202). The originally described 20 bp exon 0 transcription start site, present in both human and murine ghrelin transcripts, is indicated by a pound mark (#).

Cap analysis of gene expression (CAGE) tags average 20–21 nucleotides and are produced by large-scale sequencing of concatemers derived from the 5' ends of capped mRNA [17,18]. The CAGE method, therefore, detects the most 5' site of the mRNA transcripts – the transcription start site. Even singly, CAGE tags are considered to be reliable markers of transcription start site (TSS) locations [19]. Intriguingly, a human CAGE tag starting site (CTSS) corresponding exactly to a 106 bp exon -1 transcription start site (Fig. 3A), was found via the CAGE basic viewer. Furthermore, a 92 bp exon -1 (exon -1b, TSS2 in Fig. 2) containing transcript was amplified by 5' RACE from adipose tissue [GenBank:EF549564], leukocytes and the spleen. Sequence analysis demonstrates that it splices into a 197 base pair exon 0 (197 bp from the 3' termini of exon 0). Thus, the 100 bp conserved region, identified using Mulan and confirmed experimentally, harbours an exon -1 with two transcription start sites.

Several transcripts initiating in the 5' extended exon 0 were obtained in many of the tissues examined [GenBank:EF549565, EF549562, and EF549563] including normal human testis, stomach, adipose tissue, leukocytes and ovary (TSS 3–5, Fig. 2). While it is possible that the transcription start sites identified in exon 0 via 5' RACE represent truncated cDNA, the sites are likely to be genuine, as we found multiple CAGE tag starting sites in the putative, extended exon 0 of mouse ghrelin (Fig. 3B). While the transcription start site of the short 20 bp human exon 0 aligns with the murine start site, the TSSs of the human extended exon 0 and the putative extended murine exon 0 are quite different (Fig. 3B). This suggests that these exons have diverged significantly over time, resulting in considerable variation in their start sites, termed TSS turnover [19]. TSS turnover, with the translocation of mouse start sites compared to human start sites, occurs in a number of genes [19].

Recent studies indicate that many genes have broad transcriptional regulation with a wide distribution of proximal start sites, and not all genes are regulated by distinct start sites controlled by a TATA box [19,20]. While a putative TATA box flanks the originally described 20 bp exon 0, it appears to be a very weak start site [21,22]. The cluster of transcription start sites (TSSs) in the extended exon 0 sequence upstream of the short 20 bp exon 0 (Fig. 3B) contains no apparent TATA boxes (data not shown). Our study indicates that the ghrelin gene is broadly regulated and has many potential transcription start sites. This may allow the transcription of numerous tissue-specific and developmental stage-specific transcripts. Using *in silico *analysis coupled with RT-PCR and 5' RACE analysis, we have demonstrated that the conserved regions upstream of exon 1 (exon -1 and extended exon 0) are transcribed and correspond to novel first exons of the human ghrelin gene.

### Multiple transcripts arise from alternative splicing from exons upstream of exon 1

Expression of exon -1-containing and extended exon 0-containing transcripts were examined using RT-PCR with exon-specific sense primers (for exons -1 and extended exon 0) and with an antisense primer in the 3' terminal exon 4 of the ghrelin gene. A list of exons and exon-intron boundaries of ghrelin locus derived-transcripts identified in this and previous studies, as well as ESTs, is given in [Additional file 1].

**Figure 4:**
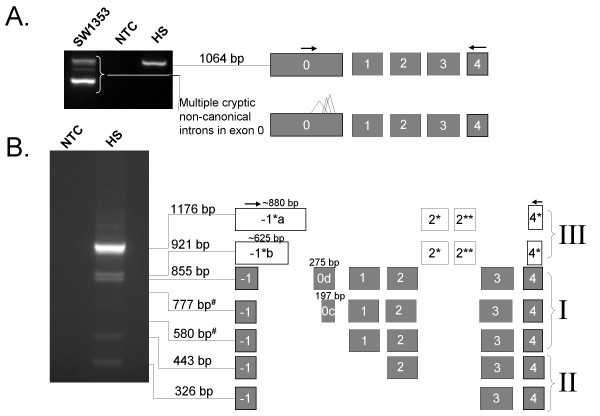
**Expression of exon -1 and extended exon 0 mRNAs in stomach and SW1353 cell line**. Exons are represented as boxes, PCR primers are indicated as arrows above exons. **A**. Amplicons spanning the extended exon 0 to exon 4 of the ghrelin gene. A major 1064 base pair amplicon, corresponding to a full-length transcript, is expressed in the human stomach (HS). The SW1353 chondrosarcoma cell line expresses a complex pattern of alternatively-spliced products, which may result from promiscuous splicing of exon 0. **B**. Exon -1 to 4 amplicons sequenced from the human stomach. Transcripts have been grouped into three major types (I, II and III). Group I amplicons include all preproghrelin exons (exons 1–4), but differ in the length of sequence upstream of exon 1 (the 5' UTR of preproghrelin). Group II transcripts contain exon -1, exon 4 and various combinations of exons downstream of exon 1 (exon 2 to 4), but lack exon 0 and 1. Group III transcripts contain alternative exon -1 and 4 (exon -1* and 4*, respectively) in addition to two novel exons (exon 2* and 2**) in intron 2 of the ghrelin gene.

Using sense primers in exon 0, a 1064 bp product that spanned exons 0 to 4 and contained a 558 bp exon 0 [GenBank:EF549569] was found in the human stomach (Fig. 4A). Moreover, primer walking with sense primers further downstream in exon 0 always resulted in bands corresponding to exon 0 and 1 (data not shown). Several PCR products were amplified from the SW1353 chondrosarcoma cell line (and these are depicted in Fig. 4A). The difference in the sizes of the transcripts results from multiple non-canonical introns in exon 0 (data not shown), and are most likely due to promiscuous splicing in the continuous tumour cell line.

**Figure 5:**
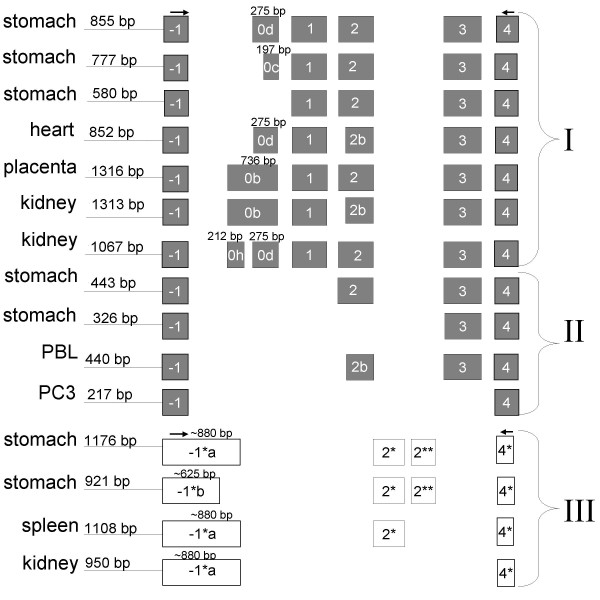
**Exon structure of exon -1 to 4 RT-PCR amplicons in human tissues and continuous cell lines**. Exons are represented as boxes, PCR primers are indicated as arrows above exons. Transcripts have been grouped into three major types (I, II and III). The tissue where an amplicon was first sequenced from is depicted on the left hand side of each transcript's exon structure. Group I are preproghrelin mRNA variants with different 5' untranslated regions (5' UTRs). Group II lack the coding region 'active core' of ghrelin (in exon 1) and contains one or more downstream exons (2–4). Group III contains the novel exons -1*, 4*, 2* and 2** in various combinations. PBL denotes leukocytes; PC3 is a human prostate carcinoma cell line. Group I: 855 bp, stomach [GenBank:EF549571];777 bp, stomach [GenBank:EF549572];580 bp, stomach [GenBank:EF549573];852 bp, heart [GenBank:EU072085];1316 bp, placenta [GenBank:EU072083];1313 bp, kidney [GenBank:EU072084];1067 bp, kidney [GenBank:EU072087]. Group II: 443 bp, stomach [GenBank:EF549574];326 bp, stomach [GenBank:EF549574];440 bp, PBL [GenBank:EU072086];217 bp, PC3 [GenBank:EF549557]. Group III: 1176 bp, stomach [GenBank:EF549568];921 bp, stomach [GenBank:EF549570];1108 bp, spleen [GenBank:EU072081];950 bp, kidney [GenBank:EU072082].

We then examined the alternative splicing of transcripts expressing exon -1 (located 2.6 kb upstream of exon 1) in human tissues and a range of human continuous cell lines. RT-PCR using sense primers to exon -1 revealed multiple transcripts in the normal human stomach [GenBank:EF549568, EF549570, EF549571, EF549572, EF549573, EF549574, and EF549575] and several other tissues and cell lines [GenBank:EF549557, EU072081, EU072082, EU072083, EU072084, EU072085, EU072086, and EU072087]. The transcripts were classified into three groups based on their sequences. The amplicons sequenced from the human stomach are depicted in Fig. 4B, while Fig. 5 summaries all exon -1 to 4 amplicons obtained in this study. The first two groups have exon structures that obey the GT/AG rule, while transcripts in the third group harbour canonical GT/AG intron splice sites in the antisense direction only.

In the first group (I), all amplicons include exon 1 to 4 which code for preproghrelin, and vary only in the length of the sequence upstream of exon 1 (the preproghrelin 5' UTR). The amplicons obtained from the human stomach [GenBank:EF549571, EF549572, and EF549573] are depicted in Fig. 4B. The 855 bp amplicon, demonstrated in the human stomach, was also observed in all cell lines examined (DU145, RWPE-1, RWPE-2, LNCaP, PC3 and SW1353, data not shown), as well as in the heart, brain, spleen, testis, salivary gland, leukocytes and bone marrow (see [Additional file 2]). As depicted in Fig. 5, sequencing of the Rapid-Scan human tissue cDNA panel also revealed a 1316 bp amplicon with a 736 bp exon 0b in the placenta [GenBank:EU072083]. Furthermore, splice variants with an alternative exon 2 splice site (hereafter termed exon 2b), which results in loss of a glutamine residue at position 14 of the mature ghrelin peptide (termed des-Gln^14^-ghrelin or ghrelin-27) [23], were also sequenced and correspond to a 1313 bp [GenBank:EU072084] amplicon from the kidney and a 852 amplicon from heart tissue [GenBank:EU072085] (Fig. 5). Interestingly, a 1067 bp amplicon with a 212 bp exon 0h initiating at the start of the 736 bp exon 0, followed by the 275 bp exon 0d (separated by a 294 bp novel intron), was found in the kidney [GenBank:EU072087]. A single nucleotide polymorphism (SNP) g.-1062G > C (nucleotide number -1 corresponds to the first nucleotide upstream of the translation start site of preproghrelin in exon 1 of the ghrelin gene) [dbSNP:rs26311] is present in base 209 of the 736 bp exon 0 (exon 0b), creating a 3' splice-site consensus sequence (CAG) [24]. This polymorphism has recently been linked to obesity and metabolic syndrome in the Korean population and is thought to influence the ghrelin promoter, ultimately increasing preproghrelin transcription efficiency [25]. Our findings raise the possibility that this SNP effects mRNA splicing, resulting in allele-specific transcription of a 736 bp (exon 0b) or a 487 bp exon 0 (the latter resulting from g.-1062C induced splicing of a 212 bp exon 0h into a 275 bp exon 0d).

The large, extended 736 bp exon 0 is extensively spliced and contains numerous non-conserved uORFs (upstream open reading frames), while exon -1 contains a single translation start site in the human sequence only (data not shown). Approximately 12% of human genes are alternatively spliced within their 5' untranslated regions [26]. Upstream open reading frames, as well as mRNA secondary structure and other motifs in 5' UTRs are known to regulate the translation of downstream major ORFs and particularly those which translate developmental genes [27]. We suggest that the alternative transcripts identified which splice into exon 1 may be a part of such a regulatory mechanism. The 20 bp exon 0 found in human stomach and thyroid medullary carcinoma TT cells [6,7] is devoid of upstream open reading frames and stable secondary structure (data not shown). As a consequence, this transcript may be more efficiently translated than the group I transcripts with exon -1 and extended exon 0 which have more extensive 5' untranslated regions.

**Figure 6:**
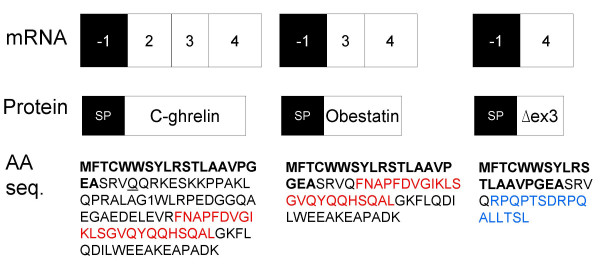
**Structure of ghrelin splice variants containing a putative signal peptide**. For each splice variant the exon structure, the predicted protein structure and amino acid sequence is depicted. A putative signal peptide (SP) in exon -1 is indicated by bold letters in the amino acid sequence. Obestatin coding sequence is depicted in red font, while an exon 3-deleted proghrelin peptide is shown in blue font. The splice variant that may encode putative C-ghrelin [GenBank:EF549574] was also expressed in the heart and spleen. Obestatin mRNA variants were obtained from the human stomach [GenBank:EF549575], while a variant that may encode an exon 3-deleted (Δex3) proghrelin peptide [GenBank:EF549557] was found in the PC3 human prostate carcinoma cell line. A mRNA variant obtained from leukocytes with a 5' truncated exon 2 [GenBank:EU072086] may encode a C-ghrelin peptide missing a single glutamine residue at position 24 (des-Gln^24^-C-ghrelin, underlined in the C-ghrelin amino acid sequence).

The second group (II) of transcripts contains splice variants which include exon -1 in various combinations with exons downstream of exon 1 (exons 2 to 4). In addition to the two 443 and 326 bp amplicons cloned from the human stomach [GenBank:EF549574, and EF549575] (Fig. 4B), we obtained a 217 base pair amplicon (EF549557) in the PC3 human prostate carcinoma cell line corresponding to a transcript that lacks exons 0–3, but contains exon -1 and exon 4, flanked by GT/AG splice junctions (Fig. 5). Furthermore, the 443 bp amplicon from the human stomach was also sequenced from the heart and spleen (data not shown) and amplicons at the expected size were observed in leukocyte and bone marrow (see [Additional file 2]). Moreover, sequencing of leukocyte-derived amplicons demonstrated an mRNA variant with a previously described 3 base pair 5' truncated exon 2 [23], exon 2b (440 bp amplicon in Fig. 5) [GenBank:EU072086]. Given that exon 1 is skipped in all group II variants, preproghrelin and the N-terminal 'active core' (Gly-Ser-Ser-(n-octanoyl)-Phe) of the ghrelin hormone [28] cannot be translated from them. Interestingly, analysis of these variants using SignalP V3.0 [25] predicts a signal peptide (MFTCWWSYLRSTLAAVPGEA) in exon -1 (with a signal peptide probability of 0.57, signal anchor probability of 0.00, and a cleavage site between position 19 and 20 which was assigned a cleavage site probability score of ~0.47). Indeed, if the signal peptide is translated, the putative peptides would be in-frame with previously reported ghrelin gene derived peptides (depicted in Fig. 6). The putative peptides encoded by these transcripts would include the sequence for C-ghrelin (ex -1, 2, 3, 4) (which also includes the coding region for obestatin [10]), the hormone obestatin alone (ex -1, 3, 4) [9], and also a novel C-terminal proghrelin peptide (exon 3-deleted proghrelin) (ex-1, 4) that is upregulated in prostate [5] and breast [3] cancer. The identification of several mRNA variants with coding sequence in-frame with an exon -1 encoded putative signal peptide strongly suggests that the signal peptide is translated and functional. For example, we have demonstrated the expression a C-ghrelin mRNA variant in human heart tissue. C-ghrelin circulates at high levels in patients with heart failure and at low levels in patients with myocardial infarction, and do not correspond with ghrelin levels [10]. In rat plasma and rat tissues, C-ghrelin levels do not appear to correspond directly with ghrelin levels [11]. Therefore, the regulation of preproghrelin and C-ghrelin could be independent and C-ghrelin could be a ghrelin gene derived hormone with distinct functions. Interestingly, a murine testis specific transcript, the ghrelin-gene derived transcript or GGDT, that codes for obestatin but not ghrelin, has previously been demonstrated and harbours a putative nuclear localisation signal [29]. While the functions of obestatin remain somewhat controversial [9,30-32], it may play a role in sleep [33], anxiety [34] and in cell proliferation [35].

### Natural antisense transcripts are transcribed from a gene on the opposite strand of ghrelin (ghrelinOS)

The third (III) group of alternative transcripts containing exon -1 and 4 that we identified in the human stomach (a 1176 bp amplicon [GenBank:EF549568] and a 921 bp amplicon [GenBank:EF549570], Fig. 4B) result from splicing of transcripts with exon -1 sequences of ~880 bp, termed exon -1*a, and ~625 bp, termed exon -1*b. These exons extend into intron -1 and are considerably larger than the 106 bp and 92 bp exon -1 sequences obtained in this study. Moreover, these splice variants also contain two novel intron 2-derived exons (exon 2* and 2**) and an alternative exon 4 (exon 4*) (Fig. 4B). As is the case in the stomach, the fragments corresponding to these unusual transcripts were observed in all cell lines examined (DU145, RWPE-1, RWPE-2, LNCaP, PC3 and SW1353, data not shown). The 1176 bp amplicon expressed in the stomach is also expressed in the heart and fetal liver (data not shown). Furthermore, an mRNA variant with a ~880 bp exon -1*a and exon 2*, but lacking exon 2** (the 1108 bp amplicon in Fig. 5) was sequenced from the spleen [GenBank:EU072081], while a 950 bp amplicon [GenBank:EU072082] harbouring exon -1*a and exon 4* only was obtained from the kidney (Fig. 5). We then determined the direction of transcription of these transcripts. GMAP [36] and manual analyses were performed using sequenced PCR products obtained in this study, as well as expressed sequence tags (ESTs) spanning at least one intron. This analysis showed that while there are no canonical GT/AG splice junctions if the variants are transcribed from the sense (ghrelin gene) DNA strand, the reverse strand contains GT/AG intron junctions. All exons demonstrated this pattern with the exception of the intron flanking exon 2* of these fragments and -1*a/b, where the splice junction is GC/AG. GC/AG is relatively rare, but the most common non-canonical splice site pair [37].

**Figure 7:**
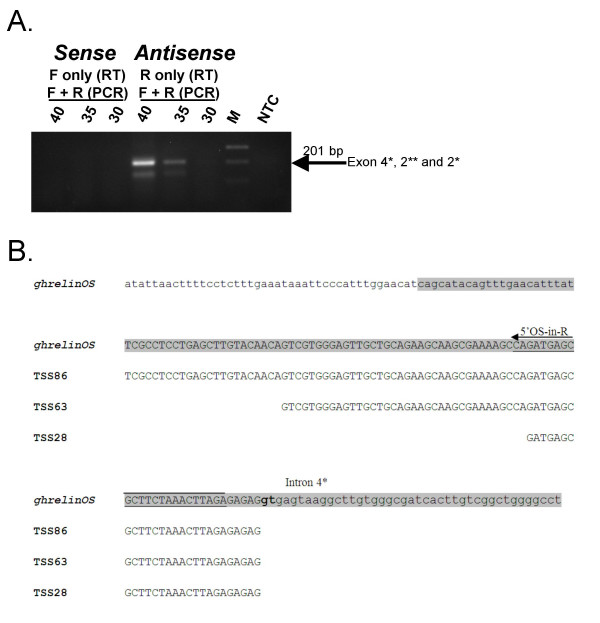
**Characterisation of ghrelin natural antisense transcripts in the normal human stomach. A**. Ethidium bromide stained agarose gel showing the verification of the candidate antisense gene ghrelinOS by orientation-specific RT-PCR in the human stomach. Antisense transcripts were amplified using a primer in exon 4* as the reverse transcription (RT) primer (R), while for the detection of sense transcripts, a primer in exon 2* was used in the RT reaction (F). The RT reactions were subjected to PCR using both the exon 2* and 4* primers (for 30, 35 or 40 cycles). NTC = no template control (water). M = MassRuler Express DNA ladder (Fermentas). **B**. Alignment of sequences derived from two 5' RLM-RACE products (TSS86 and TSS63) and a CAGE tag starting site corresponding to a 28 bp exon 4* (TSS28, T03F009D342E) with exon 4* and flanking genomic sequence. The lower case letters indicate upstream genomic or downstream intron 4* sequences. The position and sequence of the nested gene-specific 5' RACE primer (5'OS-in-R) is indicated with an arrow and underlined, respectively. For comparison, the (sense) ghrelin gene exon 4 sequence is shaded in grey.

To confirm the direction of transcription of the putative antisense transcripts, we employed strand-specific primers in reverse transcription (RT) reactions to specifically target either sense or antisense transcripts. RT-PCR analysis of human stomach cDNA (performed by combining the sense and antisense RT-primers) revealed that the target transcripts are transcribed in the antisense direction (Fig. 7A). Furthermore, Southern hybridisation, employing a nested DIG-labelled PCR probe, demonstrated a very strong signal for the antisense-direction amplicon (data not shown). The expected 201 base pair amplicon (spanning exon 4*, 2** and 2*) was isolated and sequenced to confirm its identity [GenBank:EF549558]. We have termed this antisense gene ghrelinOS (ghrelin opposite strand).

In order to firmly establish the origin of the antisense ghrelinOS mRNA transcripts, and to determine how far they extend relative to the ghrelin gene, 5' RLM-RACE using human stomach cDNA was performed. Sequencing of RACE products identified two transcription start sites (TSSs) corresponding to a 63 bp and an 86 bp exon 4* [GenBank:EF549559, and EF549560]. Furthermore, a CAGE tag starting site was identified (T03F009D342E) in the antisense direction corresponding to a 28 base pair exon 4*. The three TSSs of ghrelinOS transcripts are summarised in Fig. 7B. We did not identify any potential TATA-boxes, therefore, these findings suggest that exon 4* contains multiple TSSs, which is typical of TATA-less promoters [20].

**Figure 8:**
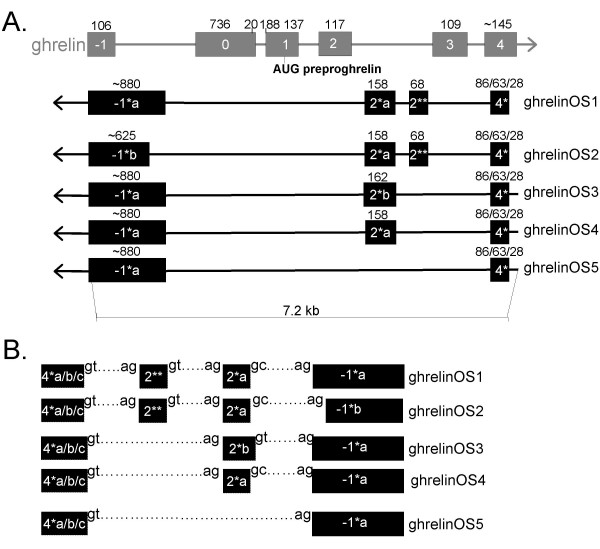
**Genomic organisation of sense and natural antisense transcripts associated with the ghrelin gene. A**. The natural antisense transcripts are shown in black and the corresponding genomic structure of the ghrelin gene in grey. The previously reported 20 bp exon 0 and extended (188 bp) exon 1 of the ghrelin gene are also shown. Exons are represented as boxes, introns as horizontal lines and sizes (bp) are indicated above each exon. The direction of transcription of each gene is indicated by arrowheads. A tilde symbol (~) in front of the size of some exons indicates that as RT-PCR primers spanned these exons, their exact ends are unknown. The antisense strand ghrelinOS transcripts overlap with the sense exon -1 and 4, but not with the exon 0–3 of the ghrelin gene. The three TSSs resulting in 86, 63 or 28 base bp exon 4* are shown. Note that it has not been determined which TSS(s) the various ghrelinOS variants employ. GhrelinOS1 contains an approximately 880 bp exon -1*a, a 158 bp exon 2*a, a short 68 base pair exon 2** and exon 4*. GhrelinOS1 was demonstrated by experimental procedures (RT-PCR) in this study and is supported by expressed sequence tag (EST) data (EST CF264800). GhrelinOS2 (demonstrated via RT-PCR in our study) is similar to ghrelinOS1, except in this variant exon -1* is ~625 bp long (exon -1*b). GhrelinOS3 (Incyte clone LIFESEQ4072309) is similar to ghrelinOS1, but lacks exon 2** while exon 2* contains a 4 bp 3' extension (exon 2*b). GhrelinOS4 is similar to ghrelinOS3, but contains exon 2*a, while ghrelinOS5 lacks exon 2* and 2** (both splice variants demonstrated in our study). **B**. Schematic organisation of the intron junctions of ghrelinOS natural antisense mRNA variants ghrelinOS1–5 described in A. All exons are flanked by GT/AG intron junctions (with the exception of ghrelinOS1, 2 and 4 (2*a to -1*a/b which is flanked by GC/AG).

Sequence analysis demonstrates that the ghrelinOS gene undergoes substantial alternative splicing, and we have identified five natural antisense transcripts (termed ghrelinOS1–5, Fig. 8A). The transcripts differ in the length of exon -1* (GhrelinOS1,3–5 *vs *2), while in the third transcript (GhrelinOS3) exon 2* is extended and exon 2** is absent. GhrelinOS4 lacks exon 2** and harbours the canonical exon 2*. Finally, GhrelinOS5 lacks both exon 2* and 2**. The exon-intron junctions of GhrelinOS transcripts are depicted in Fig. 8B. The analysis revealed no significant sequence similarity to any known gene, protein or to any long ORFs (data not shown), suggesting that these transcripts may function as regulatory, non-coding RNA [38]. Natural antisense transcripts (NATs) that are transcribed from the opposite strands of the same genomic locus are termed cis-NATs [39].

The ghrelin NATs that we have described span the non-coding 3' UTR region of exon 4 and exon -1 of mature, sense ghrelin gene-derived transcripts and overlap intron 2 sequence of ghrelin pre-mRNAs (Fig. 8A). Sense exons -1 and 4 are conserved when compared to mouse genomic sequence, while the degree of sequence similarity to exon -1* (the region not overlapping the sense exon -1), exon 2* and exon 2** appears to be very low (data not shown). Interestingly, most previously reported cis-NATs overlap with the sense transcript in their untranslated regions [39]. This appears to be the case for ghrelinOS transcript as exon -1 and 4 corresponds to 5' and 3' UTRs of sense ghrelin transcripts encoding preproghrelin.

It has been demonstrated that the mammalian genome is often transcribed from both the sense and antisense DNA strands [40]. Although the understanding of the mechanisms of action of natural antisense transcripts remains in its infancy, these transcripts have been shown to be involved in transcriptional and post-transcriptional regulation. NATs have been associated with a range of regulatory mechanisms, including transcriptional interference, RNA masking and dsRNA mediated gene-silencing via direct interaction between the sense and antisense transcripts [39,41]. Intriguingly, in a very recent study, rats were administered a short, 22 base pair ghrelin antisense oligonucleotide into the cerebrospinal fluid [42]. The antisense oligonucleotide is complementary to sequence in the rat preproghrelin 3' UTR in exon 4 (exon 4* of putative rat ghrelinOS transcripts). The study found that the antisense oligonucleotide decreased anxiety in rats (the opposite effect to ghrelin) and may act as an antidepressant [42]. We suggest that this preliminary evidence may provide a first glimpse of the function of endogenous ghrelin natural antisense transcripts.

It has been reported that ghrelin mRNA and protein levels are dissociated [43-45]. We hypothesise that this may be due to either the presence of upstream open reading frames in exons 5' to exon 1 (exon 1 harbours the preproghrelin start codon); expression of ghrelin locus derived transcripts lacking coding potential for ghrelin; or non-coding sense and/or antisense regulatory transcripts. The transcripts identified in this study may be examples of at least one of these factors. Therefore the physiological significance of each transcript species, in particular mRNA variants encoding preproghrelin, cannot be determined based on mRNA expression data alone.

## Conclusion

In the present study, we have shown that two newly identified exon regions of the ghrelin gene have the potential to generate a broad and complex transcriptional repertoire. Furthermore, a gene on the antisense strand of ghrelin, ghrelinOS, generates antisense transcripts that overlap conventional, sense ghrelin gene-derived transcripts. It would now seem imperative to perform a large-scale, thorough re-examination of the ghrelin locus in order to identify and characterise novel transcripts and peptides, as well as their function in various physiological and pathophysiological states, including obesity, depression and cancer. Such efforts may result in a reconceptualisation of the regulation and mechanisms of action of the multifunctional ghrelin peptide.

## Methods

### Bioinformatics

The identification of conserved regions in the ghrelin locus was facilitated by comparisons of the chromosomal regions spanning the human [GenBank:NT_022517], mouse [GenBank:NC_000072] and chicken (Contig12.626 of WASHUC2 draft release 2.1) ghrelin loci using Mulan (MUltiple sequence Local AligNment and conservation visualization tool) [46]. The Evolutionary Conserved Region (ECR) Browser within Mulan was used to visualise phylogenetically conserved regions of 100 bp with greater than 70% sequence identity between the ghrelin genes and 20 kb upstream of their preproghrelin translation start sites. The human ghrelin gene sequence [GenBank:NM_016362] and the 20 bp exon 0 sequence [6,7] was used as the reference sequence for annotation in Mulan. The CAGE (Cap Analysis of Gene Expression) basic viewer provided by the FANTOM (Functional Annotation of Mouse) consortium [47] was employed to locate transcription start sites in the putative first exons of the human and murine ghrelin loci. The exon-intron-structure of ghrelin locus-derived ESTs and mRNA entries identified from BLAST searches, as well as sequenced PCR amplicons obtained in this study, were analysed against the human genome (NCBI release 35) using GMAP [36]. The location of putative signal peptides of deduced amino acid sequences were predicted using the SignalP 3.0 Server with default parameters [48]. The HapMap genome browser [49] was employed to locate single nucleotide polymorphisms (SNPs) in novel ghrelin exons.

### Cell culture

The chondrosarcoma cell line SW1353 (ATCC HTB 94; a kind gift from Shea Carter, IHBI, Brisbane, Australia) was cultured in DMEM/F12 media (Invitrogen, Mount Waverley, Australia). Cultured SW1353 cells were grown in T80 or T175 flasks (Nagle Nunc International, Roskilde, Denmark) in 95% CO_2 _in a Sanyo incubator at 37°C. Prostate and/or prostate cancer derived cell lines DU145, RWPE-1, RWPE-2, LNCaP and PC3 cell lines were obtained from the American Type Culture Collection (ATCC, Rockville, MD) and cultured in RPMI1640 media (Invitrogen) as described previously [50].

### RNA extraction and reverse transcription

RNA was harvested from cultured cells at 70% confluence using RNeasy kits and on-column DNase treatment (QIAGEN, Doncaster, Australia). cDNA was synthesised in a final volume of 20 μl from 3 μg total RNA, from human stomach (FirstChoice, Ambion, Austin, TX) and cell lines, using Transcriptor reverse transcriptase (Roche, Castle Hill, Australia), 20 U RNasin Plus RNase Inhibitor (Promega, Annandale, Australia) and, unless otherwise indicated, oligo(dT)_18 _primers (Proligo, Armidale, Australia) according to the manufacturer's instructions.

### RT-PCR verification of putative ghrelin exons identified by comparative genomics

The existence of putative exon -1 and extended exon 0 were verified by RT-PCR employing primers spanning the putative exons (EX-1-F/R and V0-F/R, Table 1). RT-PCR was performed in a total reaction volume of 50 μl containing 1 × PCR buffer, 0.2 mM deoxynucleotide triphosphates, 1.5 mM MgCl_2_, 0.2 μM primers, 2 μl cDNA and 1 unit of Platinum Taq DNA Polymerase (Invitrogen) on a PTC-200 thermal cycler (MJ Research, Watertown, MA) according to the manufacturer's instructions. All PCR primers were synthesised by Proligo. PCR products were purified from agarose gels using the High Pure PCR Product Purification Kit (Roche). All products were subcloned into pGEM-T Easy (Promega) and sequenced by the Australian Genome Research Facility (Brisbane, Australia) using the ABI PRISM BigDye Terminator Cycle Sequencing Kit v3.1 protocol (Applied Biosystems, Foster City, CA).

**Table 1:** Designations and sequences of primers used in RT-PCR

**Primer name**	**Primer sequence (5'-3')**	**Exon**	**Ta (°C)**	**PCR Cycles**
V0-F	GAACATTTTTAGTTCCCAAGGAATG	0		
V0-R	CTCTCTGGTGTTCAGGGCTC	1	62	40
EX-1-F	AAGATAACACAGCTTTGCACAG	-1		
EX-1-R	CTCTGGTGTTCAGGGCTCAG	1	59	40
Sure-F	TTCGTCACTCCGTGAATCAG	N/A		
Outer-R	TGGCGTTTGTTCTAAACCAG	0	59 → 57	25 (10 → 15)
Inner-R	GGCTGATGTACATTCCTTGG	0	59	35
Exon-1-Full-F	GTGGATGTTTACTTGCTGGTG	-1/-1* #		
Exon-1-Full-R	GTTTGAACATTTATTCGCCTCC	4/4* #	61	35, 40
V-long-ex0-F	GAGGATGATCAGGAGCAGTC	0		
V-long-ex0-R	CAGTTTGAACATTTATTCGCCTCC	4	59	40
B2MG-F	TGAATTGCTATGTGTCTGGGT	2		
B2MG-R	CCTCCATGATGCTGCTTACAT	3	55	25
cRNA-exon-1-F	CCCAAAGATAACACAGCTTTGCAC	-1		
cRNA-exon-1-R	CTAATACGACTCACTATAGGGAGATGGACCCTGGAGGCCTCT	-1	64	30
CF264800-F	ATGAGCGCTTCTAAACTTAGAG	4/4*		
CF264800-R	GCTCCTGTTTCCTAAGATGTC	2*	59	30, 35, 40
ex2*-nested-South-F	GCATTTGCCTCAGCGGT	2*		
ex2*-nested-South-R		2*	59	30
5'adapter-out-F	CTGATGGCGATGAATGAACAC	N/A		
5'OS-out-R	TCTCTAAGTTTAGAAGCGCTC	4*	59	35
5'adapter-in-F	ATGAATGAACACTGCGTTTGCT	N/A		
5'OS-in-R	TCTAAGTTTAGAAGCGCTCATCTG	4*	61	35

T7 promoter sequence in primer cRNA-exon-1-R is underlined. Primers in exon -1 and 4 also span antisense exons (-1* and 4*, respectively) as indicated where appropriate (#).

### 5' RACE validation of novel first ghrelin exons

5' RACE was performed with primers specific to extended exon 0 using a Sure-RACE kit (OriGene, Rockville, MD) according to the manufacturer's instructions. Samples of cDNAs from 22 adult human tissues (brain, heart, kidney, spleen, liver, colon, lung, small intestine, muscle, stomach, testis, placenta, pituitary, thyroid gland, adrenal gland, pancreas, ovary, uterus, prostate, leukocytes (PBL), adipose tissue, and mammary gland) and two fetal tissues (brain and liver) were challenged. First round PCR was performed with an adapter-specific sense primer and an exon 0-specific antisense primer (Sure-F and Outer-R, Table 1). PCR products were diluted and used in a secondary, hemi-nested PCR with a gene-specific antisense primer (Inner-R, Table 1).

### Isolation of alternatively spliced mRNAs

In order to investigate the patterns of alternative splicing of the putative first exons, RT-PCRs were performed employing an antisense primer in the 3' terminal exon 4 and sense primers in exon -1 or extended exon 0 (Exon-1-Full-F/R and V-long-ex0-F/R in Table 1, respectively). The integrity of the cDNA was confirmed by RT-PCR for β2 microglobulin using intron-spanning primers (B2MG-F/R, Table 1). Human stomach and the SW1353 chondrosarcoma cell line were examined to investigate alternative splicing of extended exon 0 transcripts. RT-PCR of exon -1 was performed using mRNA from the SW1353 chondrosarcoma cell line and from five prostate and prostate cancer cell lines (DU145, RWPE-1, RWPE-2, LNCaP and PC3). To verify their identity, exon -1 to 4 specific RT-PCR products amplified from the cell lines were transferred to a positively charged nylon membrane (Roche) for Southern analysis. The membrane was hybridised at 48°C with an antisense digoxigenin (DIG) labelled cRNA probe specific to exon -1 synthesised from a 5' RACE PCR product (cRNA-exon-1-F/R, Table 1) and detected as described by the manufacturer (Roche). PCR reagents, purification, cloning and sequencing were performed as described above.

To further investigate the expression profiles of exon -1 to 4 containing transcripts in various normal adult and fetal human tissues, RT-PCR was employed using Rapid-Scan gene expression panels (OriGene) according to the manufacturer's instructions. Rapid-Scan panels include duplicate 96-well PCR plates containing first-strand cDNA synthesised from poly(A)^+ ^RNA from 24 human tissues (brain, heart, kidney, spleen, liver, colon, lung, small intestine, muscle, stomach, testis, placenta, salivary gland, thyroid gland, adrenal gland, pancreas, ovary, uterus, prostate, skin, leukocytes (PBL), bone marrow, fetal brain, fetal liver) serially diluted over a 4-log range (1000X-1X), where the lowest concentration (1X) is approximately 1 pg cDNA. The panels are normalised to β-actin allowing an assessment of the relative abundance of exon -1 to 4 splice variants by limiting the number of PCR cycles. PCR, cloning and sequencing was performed as above, except that the samples were subjected to 35 cycles of PCR in a total reaction volume of 25 μl.

### Strand-specific RT-PCR

To verify the orientation of putative antisense transcripts, total stomach RNA was reverse transcribed using primers specific for sense or antisense transcripts (CF264800-F/R in Table 1) and subjected to PCR combining both reverse transcription primers. To verify the identity of the transcripts, a nested DIG-labelled (Roche) PCR probe (ex2*-nested-South-F/R, Table 1) was synthesised from cloned sequence and employed in Southern hybridisation at 42°C, as described previously [50]. In addition, a PCR product at the expected size of an amplicon corresponding to a putative antisense EST [GenBank:CF264800] was eluted from agarose gels, reamplified and subsequently purified and sequenced as described above.

### 5' RACE analysis of antisense transcription start sites

To characterise the 5' end of the putative antisense RNAs, 5' RACE was undertaken using FirstChoice RLM-RACE-Ready human stomach cDNA (Ambion) according to the manufacturer's instructions. The first round PCR was performed with an adapter-specific sense primer and an exon 4* specific antisense primer (5'adapter-out-F and 5'OS-out-R in Table 1, respectively). PCR product (1 μl) was used in a secondary, nested PCR with a gene specific primer in exon 4* (5'adapter-in-F and 5'OS-in-R, Table 1). PCRs were performed in a total reaction volume of 50 μl using Platinum Taq Polymerase High Fidelity (Invitrogen) according to the manufacturer's instructions.

## Authors' contributions

IS conceived and designed the study and carried out the experiments. CC, ACH and LKC participated in its design and coordination. All authors participated in interpreting the data, writing the manuscript, and read and approved the final manuscript.

## Supplementary Material

Additional file 1 Compilation of exons and exon-intron boundaries of ghrelin locus derived transcripts. This is a PDF file listing exons and intron boundaries of ghrelin locus derived transcripts. Exon and intron sizes (bp) are indicated, while 5' and 3' splice sites are shown with exonic sequences denoted by uppercase and intronic sequences by lower case letters. Experimental evidence and/or external references for each exon are shown. Exons of endogenous natural antisense transcripts derived from the antisense strand of the ghrelin gene, ghrelinOS, are underlined. A pound sign (#) indicates that, although an RT-PCR primer spanned the exon, its exact terminal sequence(s) is/are unknown. Note that the 212 bp exon 0 (exon 0h) initiates at the start of the 736 bp exon 0, while all other exon 0 sizes are numbered from the 3' terminus of the 20 bp exon 0. Click here for file

Additional file 2 Ethidium bromide stained agarose electrophoresis of exon -1 to 4 RT-PCR amplicons of OriGene human tissue Rapid-Scan cDNA panel. This is a PDF file showing the expression profile of exon -1 to 4 amplicons in 24 human tissues. The panel consists of normalised cDNA from 24 tissues or embryo stages, serially diluted over a 4-log range (1000X–1X), where the lowest concentration (IX) is approximately 1 pg. Lane 1: placenta; 2: testis; 3: stomach; 4: muscle; 5: small intestine; 6: lung; 7:colon; 8: liver; 9: spleen; 10: kidney; 11: heart; 12: brain; 13: fetal liver; 14: fetal brain; 15: bone marrow; 16: leukocytes (PBL); 17: skin: 18: prostate; 19: uterus; 20: ovary; 21: pancreas; 22: adrenal gland; 23: thyroid gland; 24: salivary gland. Sequenced amplicons, as well as their sizes (in base pairs), are indicated by a star (*) symbol adjacent to the band. An amplicon at the expected size (580 bp) of a previously sequenced amplicon is indicated by a pound (#) mark in lane 4. Amplicons of 1108 bp (sequenced from the spleen, lane 9) and 1067 bp (sequenced from the kidney, lane 10) are similar in size and cannot be adequately resolved on the agarose gel, and are therefore omitted from the figure. A particularly complex splice pattern can be seen in the spleen (lane 9), kidney (lane 10), heart (lane 11), bone marrow (lane 15) and prostate (lane 18). M = MassRuler Express DNA ladder (Fermentas). Click here for file
