# Supplementary material for: Revised genomic structure of the human ghrelin gene and identification of novel exons, alternative splice variants and natural antisense transcripts
Source: BMC Genomics. 2007 Aug 30;8:298. doi: 10.1186/1471-2164-8-298 (PMC2014779; doi:10.1186/1471-2164-8-298)
Supplement: Additional file 1 — Compilation of exons and exon-intron boundaries of ghrelin locus derived transcripts. This is a PDF file listing exons and intron boundaries of ghrelin locus derived transcripts. Exon and intron sizes (bp) are indicated, while 5' and 3' splice sites are shown with exonic sequences denoted by uppercase and intronic sequences by lower case letters. Experimental evidence and/or external references for each exon are shown. Exons of endogenous natural antisense transcripts derived from the antisense strand of the ghrelin gene, ghrelinOS, are underlined. A pound sign (#) indicates that, although an RT-PCR primer spanned the exon, its exact terminal sequence(s) is/are unknown. Note that the 212 bp exon 0 (exon 0h) initiates at the start of the 736 bp exon 0, while all other exon 0 sizes are numbered from the 3' terminus of the 20 bp exon 0. [file 1471-2164-8-298-S1.pdf]

Additional File 1: Compilation of exons and exon-intron boundaries of ghrelin locus derived transcripts. Exon and intron sizes (bp) are indicated, while 5' and 3' splice sites are shown with exonic sequences denoted by uppercase and intronic sequences by lower case letters. Experimental evidence and/or external references for each exon are shown. Exons of endogenous natural antisense transcripts derived from the antisense strand of the ghrelin gene, ghrelinOS, are underlined. A pound sign (#) indicates that, although an RT-PCR primer spanned the exon, its exact terminal sequence(s) is/are unknown. Note that the 212 bp exon 0 (exon 0h) initiates at the start of the 736 bp exon 0, while all other exon 0 sizes are numbered from the 3' terminus of the 20 bp exon 0.

Exon	Size (bp)	5' splice site	3' splice site	Intron size (bp)	Experimental evidence or external reference
-1a	106	First exon	TCCAGgtaag	1390 to exon 0b<br>1851 to exon 0c<br>1929 to exon 0d<br>2632 to exon 1a<br>2963 to exon 2a<br>2966 to exon 2b<br>6029 to exon 3<br>6947 to exon 4	5' RACE, CAGE (T03R009D4FFF) and RT-PCR
-1b	92	First exon	TCCAGgtaag	1390 to exon 0b<br>1851 to exon 0c<br>1929 to exon 0d<br>2632 to exon 1a<br>2963 to exon 2a<br>2966 to exon 2b<br>6029 to exon 3<br>6947 to exon 4	5' RACE and RT-PCR
-1*b	~625	tgcagGAGGG	Terminating exon		RT-PCR#
-1*a	~880	tccagGAAAA	Terminating exon		RT-PCR# and ESTs CF264800 (brain glioblastoma), LIFESEQ4072309 (kidney) and CF121451 (CNCAP(3)T-225 prostate cell line)
0a	20	First exon	TGCAGgtaag	506 to exon 1	
0b	736	tccagGTCCA			5' RACE, RT-PCR
0c	197	cccagCAATT			5' RACE, RT-PCR
0d	275	accagGGCCA			RT-PCR#
0e	498	First exon			5' RACE
0f	405	First exon			5' RACE
0g	368	First exon			5' RACE
0h	212	tccagGTCCA	GGCAGgtggg	294 to exon 0d	RT-PCR#
1b	188	First exon	TCCAGgtgag	0 to exon 1	
1a	137	cccagGCCCA	TCCAGgtgag	194 to exon 2a<br>197 to exon 2b	
2a	117	tccagCAGAG	TCCGGgtcgg	2934 to exon 3	
2b	114	agcagAGAAA	TCCGGgtcgg	2934 to exon 3	, RT-PCR
2*a	158	ttaagGAGAG	TGCAGgcaag	3965 to exon -1*a<br>4220 to exon -1*b	RT-PCR, ESTs CF264800 and CF121451
2*b	162	ttaagGAGAG	GGCAAgtgag	3961 to exon -1*a	LIFESEQ4072309
2**	68	tacagGGAGA	TTTGGgtaag	463 to exon 2*a	RT-PCR, ESTs LIFESEQ4072309 and CF264800
3	109	cctagTTCAA	CAAAGgtgag	809 to exon 4	
4	~145	tccagAGGCC	Terminating exon		#
4*a	86	First exon	GAGAGgtgag	1560 to exon 2**<br>2091 to exon 2*a/b<br>6214 to exon -1*a	5' RACE, RT-PCR, ESTs LIFESEQ4072309 and CF264800
4*b	63	First exon	GAGAGgtgag		
4*c	28	First exon	GAGAGgtgag		CAGE (T03F009D342E)

References

1. Kanamoto N, Akamizu T, Tagami T, Hataya Y, Moriyama K, Takaya K, Hosoda H, Kojima M, Kangawa K, Nakao K: **Genomic structure and characterization of the 5'-flanking region of the human ghrelin gene**. *Endocrinology* 2004, **145**(9):4144-4153.
2. Nakai N, Kaneko M, Nakao N, Fujikawa T, Nakashima K, Ogata M, Tanaka M: **Identification of promoter region of ghrelin gene in human medullary thyroid carcinoma cell line**. *Life Sci* 2004, **75**(18):2193-2201.
3. Wajnrajch M, Ten I, Gertner J, Leibel R: **Genomic Organization of the GHRELIN Gene**. *Journal of Endocrine Genetics* 2000, **1**:231-233.
4. Hosoda H, Kojima M, Mizushima T, Shimizu S, Kangawa K: **Structural divergence of human ghrelin. Identification of multiple ghrelin-derived molecules produced by post-translational processing**. *J Biol Chem* 2003, **278**(1):64-70.
