# Supplementary figures and images for: Revised genomic structure of the human ghrelin gene and identification of novel exons, alternative splice variants and natural antisense transcripts
Source: BMC Genomics. 2007 Aug 30;8:298. doi: 10.1186/1471-2164-8-298 (PMC2014779; doi:10.1186/1471-2164-8-298)

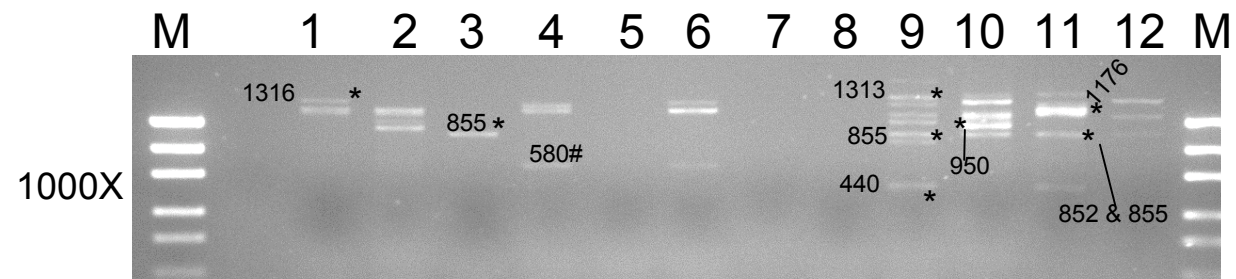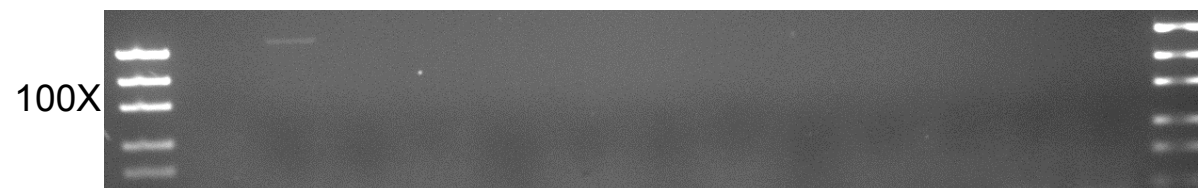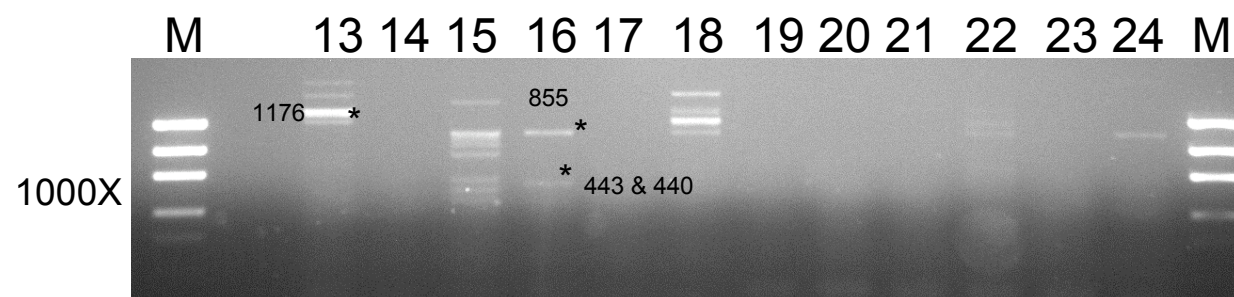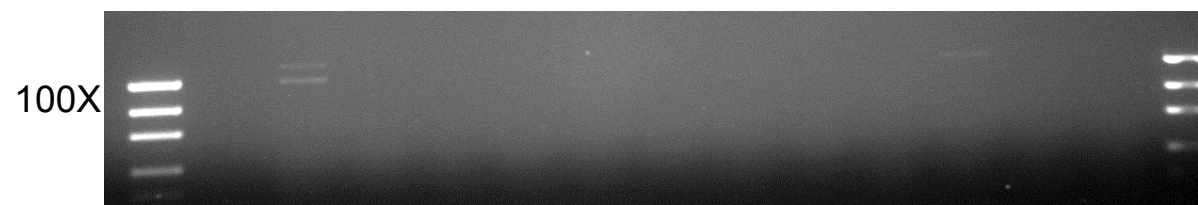

Supplement: Additional file 2 — Ethidium bromide stained agarose electrophoresis of exon -1 to 4 RT-PCR amplicons of OriGene human tissue Rapid-Scan cDNA panel. This is a PDF file showing the expression profile of exon -1 to 4 amplicons in 24 human tissues. The panel consists of normalised cDNA from 24 tissues or embryo stages, serially diluted over a 4-log range (1000X–1X), where the lowest concentration (IX) is approximately 1 pg. Lane 1: placenta; 2: testis; 3: stomach; 4: muscle; 5: small intestine; 6: lung; 7:colon; 8: liver; 9: spleen; 10: kidney; 11: heart; 12: brain; 13: fetal liver; 14: fetal brain; 15: bone marrow; 16: leukocytes (PBL); 17: skin: 18: prostate; 19: uterus; 20: ovary; 21: pancreas; 22: adrenal gland; 23: thyroid gland; 24: salivary gland. Sequenced amplicons, as well as their sizes (in base pairs), are indicated by a star (*) symbol adjacent to the band. An amplicon at the expected size (580 bp) of a previously sequenced amplicon is indicated by a pound (#) mark in lane 4. Amplicons of 1108 bp (sequenced from the spleen, lane 9) and 1067 bp (sequenced from the kidney, lane 10) are similar in size and cannot be adequately resolved on the agarose gel, and are therefore omitted from the figure. A particularly complex splice pattern can be seen in the spleen (lane 9), kidney (lane 10), heart (lane 11), bone marrow (lane 15) and prostate (lane 18). M = MassRuler Express DNA ladder (Fermentas). [file 1471-2164-8-298-S2.pdf]
